# Collaboration cognizance: Development of a self-assessment tool to measure intra-professional collaborative practices (IPCP) in postgraduate medical residents at tertiary care hospitals

**DOI:** 10.1186/s12909-024-05759-7

**Published:** 2024-07-19

**Authors:** Ayesha Anwar, Rahila Yasmeen, Rehan Ahmed Khan

**Affiliations:** 1 Department of Dermatology, Pak Emirates Military Hospital Rawalpindi, Rawalpindi, Pakistan; 2 MHPE Fellow at Riphah International University, Islamabad, Pakistan; 3 Dean Riphah Academy of Research & Education, Riphah International University, Islamabad, Pakistan; 4 Dean Riphah Institute of Assessment, Riphah International University, Islamabad, Pakistan

**Keywords:** Collaboration, Intra-professional collaboration, Residents, Self-assessment instrument, Validity and reliability

## Abstract

**Background:**

The ever-evolving healthcare system of today demands physicians who steer their roles as treatment providers, managers and collaborators. Professionals are highly interdependent due to increased complexity of health problems and risk of errors increases with transitions in care. In hospitals, the main workforce is postgraduate residents; therefore, intraprofessional collaboration amongst residents is essential. Several instruments are available to evaluate interprofessional collaboration amongst physicians, nurses and hospital teams but none specifically assessed intra-professional collaborative practices amongst residents working in tertiary care hospitals in multi-disciplinary teams. This study intends to develop and validate an instrument to self-assess intraprofessional collaborative practices in postgraduate residents undergoing residency in multiple specialties at tertiary care hospitals.

**Approach:**

This study on Instrument Development employed mixed method study design executed in two phases. In phase 1, six domains of intraprofessional collaborative practices were identified from literature and 35 items were developed. Fifteen experts participated in qualitative content validation and provided comments. To establish content validity in phase 2, content validity index (CVI) and content clarity average (CCA) were assessed by seventeen experts. Response process validity was established by cognitive interviewing of 5 postgraduate residents. Pilot testing was done on a sample of 407 residents. Cronbach’s alpha was determined, and confirmatory factor analysis established construct validity.

**Results:**

During phase 1, items were modified based on qualitative feedback from 15 experts. In round 2, CVI and CCA were determined based on responses of 17 experts. The items having an I-CVI greater than 0.90 were accepted and six items underwent modifications as their I-CVI fell between 0.78 and 0.90. Similarly, four items with a CCA of less than 2.4 were modified to increase clarity. Cognitive interviews of participants on 30 items resulted in the deletion of 1 item and changes in 5 items. The final instrument had 29 items categorized under six constructs. All items had good factor loadings during CFA, so none was deleted. Cronbach’s Alpha α was 0.937.

**Conclusion:**

Intraprofessional collaborative practices in residents is a valid and reliable self-assessment tool comprising 29 items measuring six constructs. It may be used by residents to assess their collaborative practices and incorporated in curricula to help develop collaborative practices and their assessment during training of postgraduate residents.

**Supplementary Information:**

The online version contains supplementary material available at 10.1186/s12909-024-05759-7

## Introduction

Globally, the healthcare system is undergoing tremendous evolution and role of healthcare professionals particularly physicians is undergoing a continuous transformation to keep up with trends in technology, financial constraints and new societal and scientific standards [1]. Physicians of today need to balance their roles as health providers as well as effective managers and collaborators. Their working lives involve constant interactions set in collective environments. These interactions are the basis of a need for collaboration. According to Oxford Advance Learner Dictionary online [2], collaboration is defined as “the act of working with another person or group of people to create or produce something.” For health professionals, it implies the idea of collective action based on trust and harmony and oriented toward a common goal [3]. It incorporates respect, communication and understanding other healthcare disciplines to improve quality of patient care [4]. Healthcare professionals need to cooperate and share responsibility for decision making to formulate and carry out effective patient care plans [5]. Therefore, a better understanding of interprofessional as well as intraprofessional collaboration is need of the hour. There are a number of hurdles which are encountered while trying to foster a collaborative environment. These include time constraints, a perceived loss of autonomy, lack of trust and knowledge about skill of professionals in other disciplines leading to a clash in perceptions [6].

In hospitals, the main workforce is postgraduate residents undergoing their training. Therefore, intraprofessional collaboration (intra PC) among residents is important to reduce adverse events in patient care, maintenance of quality of care and preservation of continuity of care [7]; thus, requiring attention and a deliberate effort for inculcation.

The new healthcare environment post COVID-19 pandemic presents a great opportunity to develop the next generation of healthcare professionals, appropriately trained for a seismic shift in how high-quality healthcare will be delivered in the future [8]. Healthcare professionals are trained and socialized to adopt a discipline-based approach towards patients and their treatment. Each discipline has strong theoretical and discipline-based frameworks with rigid professional boundaries. This forms the bases of the professional system. Implementing a logic of collaboration rather than a logic of competition is required to change this paradigm [9].

However, in practice, a lot still needs to be done to incorporate collaborative practices amongst postgraduate residents who are the actual backbone in the hospital. In order to incorporate collaborative practices, it is essential that they be measured. Also, a lot of work has been done in interprofessional collaboration but intraprofessional collaboration has been relatively less explored. Various instruments have been constructed to measure personal insights into collaboration, collaborative attitudes in healthcare teams etc. [10] but no instrument is there to assess intraprofessional collaborative practices amongst residents. This research is an endeavor to take a small step towards this huge task of incorporating intraprofessional collaborative skills in our postgraduate residents.

The main objective of our study is to develop and validate an instrument to measure Intraprofessional Collaborative Practices (IPCP) in postgraduate residents of different specialties working at tertiary care hospitals in Pakistan. To address this, the following research questions are relevant: (a) What are the key constructs/domains of intraprofessional collaborative practices amongst postgraduate medical residents based on healthcare collaboration models? (b) How to develop the items to self-assess the domains of intraprofessional collaborative practices in postgraduate medical residents? And (c) How to validate the instrument to assess intraprofessional collaborative practices in postgraduate medical residents?

Theoretical frameworks help us understand the complexity of phenomenon. The group behaviour of collaboration is elaborated by several models of collaboration. With respect to the healthcare profession, there are three relevant theoretical models that endeavor to elaborate collaborative behaviors and practices. These are the models by Sullivan [3], D’Amour [9] and Bronstein [11]. The constructs underlying key determinants of collaboration and their measurement are described in these models [11]. The D’Amour Model is based on an analysis of 17 papers based on collaboration in healthcare teams. The identified attributes of collaboration from the D’Amour Model which included a shared healthcare ideology, partnerships and networking, interdependency and an equilibrium in power dynamics served to guide the main constructs of our instrument [9].

## Methodology

### Study design & settings

This study is a mixed method research that employed both qualitative and quantitative data collection and analysis. The study followed the steps of instrument development model with exploratory sequential design. In this design first qualitative data is gathered to explore a phenomenon, and then quantitative data is collected to explain relationships found in the qualitative data [12]. Ethical approval for the research was sought from the Ethical Review Committee of Riphah University, Reference no. Appl #: Riphah /IRC/23/3032. The study involved an input from experts in all major medical specialties as well as medical educationists and post graduate residents working in multiple fields at various tertiary care hospitals of Pakistan. The study was conducted in two major phases according to the steps of questionnaire development as elaborated in AMEE guide 87 [13].

**Fig. 1:**
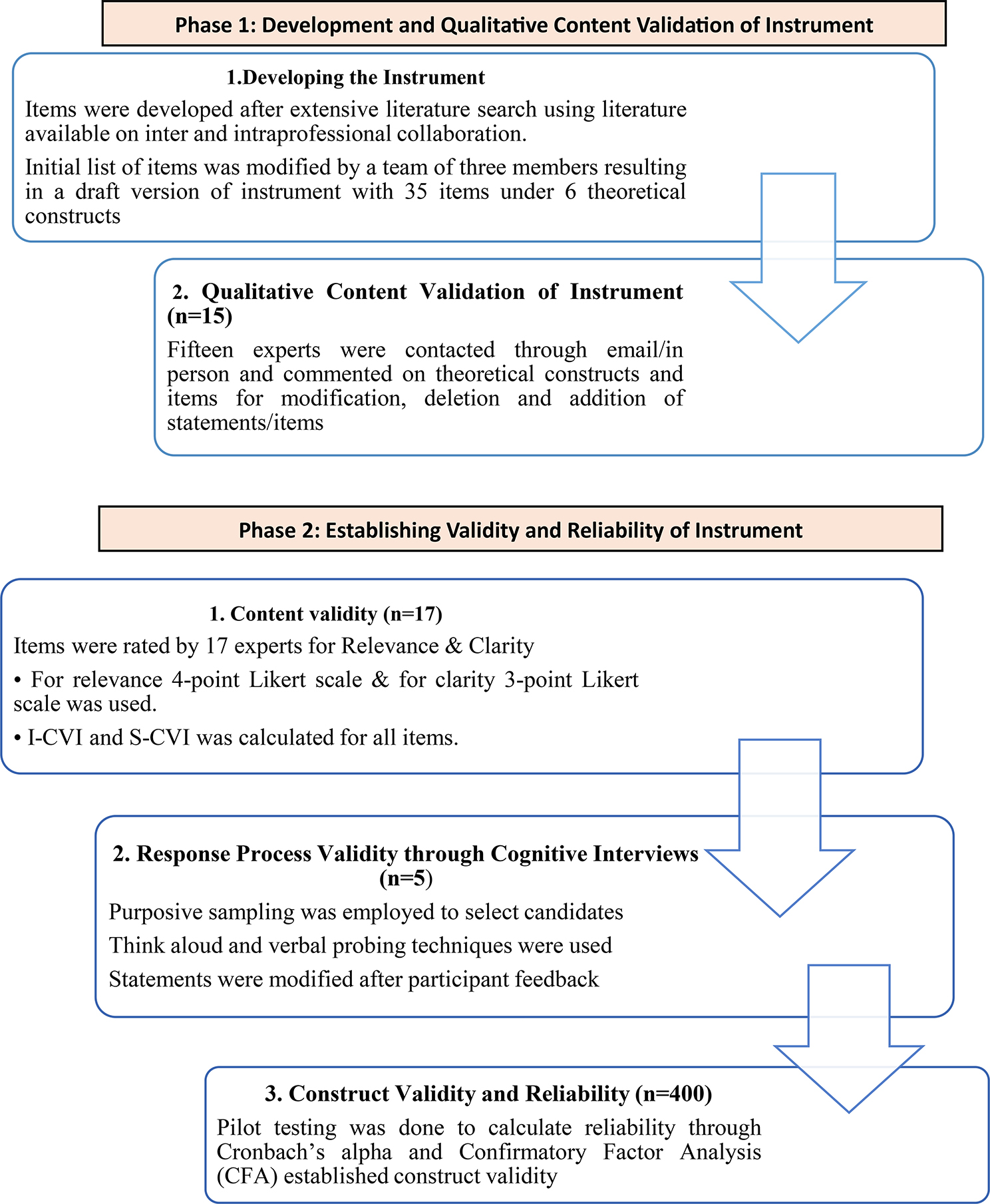
Phases of study for development and validation of Instrument

All participants were given a brief description of the study rationale and process with details available on request and consent was taken prior to qualitative and quantitative rounds of expert validation of the instrument, cognitive pre-testing and factor analysis. Participant confidentiality was ensured at all times during different steps of the research process. The study was carried out in two phases (Fig. 1).

### Phase 1: Item development and qualitative content validation

In the first phase of development, we tried to find answers to our first two research questions pertaining to the themes of intraprofessional collaboration and item development.

- **Themes identification & item development**: For development of the initial draft instrument, literature on intraprofessional collaboration as well as tools of interprofessional collaboration in healthcare were analyzed and studied. The theoretical models of collaboration especially the D’Amour Model served as the basis of theme identification [9]. Since collaboration is now accepted as one of the main physician competencies, Collaborative Competency Frameworks in Literature were studied. A total of six themes were identified from the Canadian Interprofessional Health Collaborative (CIHC) framework [14], NHS Clinical Leadership Competency Framework [15], Core competencies for interprofessional collaborative practice (IPEC)-2016 [16] and ACGME competencies for PGs [17]. An integrative review on intraprofessional collaboration competencies amongst physicians was also used in theme identification [18]. Systematic reviews on tools of interprofessional collaboration [10] were identified and the tools related to physicians working in teams were explored for item development.

A total of 35 items were constructed in the form of statements, and response anchors on a 5-point Likert scale were selected to correspond to the statements. The constructs were clearly defined for better understanding of the experts (Table 1).

**Table 1:** Constructs of Intraprofessional Collaborative Practices and their definitions

Construct	Definition
1. Roles/responsibilities for Intraprofessional collaborative practice	• Understanding, working within, and maintaining a clear distinction among duties, roles, and responsibilities
2. Intraprofessional collaborative communication	• Responsive and responsible communication to support a team-based approach
3. Intraprofessional collaborative team-based care and networking	• Use team dynamics’ relationship-building concepts to achieve success as a team.
4. Values/ethics for intraprofessional collaborative practice	• Cooperate with team members in a way that upholds a culture of respect and common values
5. Sharing of mutual knowledge for IPCP	• Being able to recognize, comprehend, and share knowledge with potential partners in collaboration
6. Intraprofessional collaborative leadership	• The ability to demonstrate effective leadership skills in a team to bring about collaboration

### a) Qualitative content validation by experts

Since main theoretical constructs were identified from various competency frameworks in literature, therefore, the step of focus group was omitted, and qualitative expert feedback was incorporated for appropriate item construction [13].

#### Materials & procedure

Using maximal variation sampling a panel of fifteen experts were approached and requested to participate in the study. These included medical educationists, heads of departments and supervisors of multiple specialties (Medicine & Allied, Surgery & Allied, Gynecology and Pediatrics) with more than five-years’ experience of supervisorship and healthcare administrators working in multidisciplinary teams. Two postgraduate residents were also included as representatives from the population being studied [13].

Instrument version 1 was sent to the identified experts for their analysis of theoretical constructs and items in terms of adjustment, removal, and addition of items. The instrument with thirty-five items grouped in six themes was sent through email as a word document and reminders were sent after one week. Hard copy was given to some of the experts and face-to-face input was also taken.

### Data analysis

The principal investigator analyzed the feedback of the experts and organized all the comments on various items of the instrument. The items were modified based on the criteria, (a) relevance of item to construct, (b) ease of understanding, (c) remove duplicate/difficult terms, (d) remove double barrel statements and (e) remove errors in spelling and grammar [19]. The principal investigator, supervisor and another team member analyzed the feedback independently and final decision on modification or deletion of the items was reached after discussion and agreement (Annexure I- qualitative expert feedback).

### **Phase II: Establishing validity & reliability of the instrument**

The next phase incorporated steps for establishing the validity and reliability of the instrument, which was our third research objective.

### a) Content validity

#### Participants & procedure

A total of 25 experts were invited for this step of the study out of which 17 responded. A content validation form as a word document with a summary of research and informed consent was emailed to 25 experts. This form (the second version of the tool/instrument) had a total of 31 items under six constructs. Experts were reminded on WhatsApp/email after 1 week and responses were documented from 17 experts within a fortnight. The experts were invited to assess each item based on its relevance to the instrument and clarity. A four point-Likert scale was used for relevance: Very relevant (VR) was graded as 4, quite relevant (QR) as 3, somewhat relevant (SR) as 2 and not relevant (NR) as 1. For clarity, a 3-point Likert scale was used: Very clear = 3, item needing revision = 2 and not clear = 1.

#### Data analysis

We calculated content validity index of individual items (I-CVI) as well as scale (S-CVI). The number of experts in agreement was divided by the total number of experts to calculate I-CVI. The number 1 was assigned to ratings of 3 or 4 on the relevance scale and 1 or 2 was assigned a value of 0. The 1s for each item were counted and divided by the number of experts to calculate I-CVI (*n* = 17) [19]. The average of CVI scores of all the items gave the S-CVI [20]. Items having, I-CVI of ≥ 0.90 were incorporated, items with I-CVI falling amid 0.78–0.90 were altered and items with I-CVI ≤ 0.78 were eliminated [19].

Content clarity average (CCA) was calculated from an expert ranking of statements on a 3-point Likert scale. CCA for individual items was calculated as the sum of all values for the item and dividing the sum by the number of experts. Items with CCA values above 2.4 (80%) were marked as very clear [19]. The details are available as Annexure II & III.

### b) Response process validity

Response process validity is to establish if the intended participants understand what is being asked by the researcher in the instrument and if the instrument is fulfilling its purpose [21]. It was done using cognitive pre-testing, a qualitative method involving interviews of participants.

#### Participants & procedure

A total of five participants were selected for the interviews. These included post graduate residents undergoing FCPS part-II training in Medicine, Surgery, Pediatrics and Gynecology at a tertiary care hospital in Rawalpindi. Residents with more than two years of training and familiar with working in multi-disciplinary environments were selected.

A pilot interview was conducted face-to-face with 1 co-investigator to detect any potential problems encountered during verbal probing and think-aloud techniques. A voice recorder application on mobile phone was used to record the interviews. Proactive probes and questions were developed using this test interview and time duration to complete one interview was also determined.

After an informed consent participants were explained the procedure and given instructions to read the items clearly and rephrase the statements in their own words to show their understanding of its meaning. Simultaneous verbal probing of participants using scripted probes was done by the investigator [22].

In the third version, the tool comprised 31 items divided into six constructs. Researcher’s bias was reduced by the presence of one co-investigator during each interview, thus avoiding questions and analysis limited to the principal investigator’s point of view. The interviews were audio recorded with participant’s consent for later analysis. The average interview lasted about 20–25 min.

#### Data analysis

Audio-recorded interviews were transcribed, analyzed, and memos were created. Finally, predefined codes were used to categorize memos: (a) items with no issues, (b) items with minor issues and (c) items with major problems [23].

The coding was done by two reviewers independently to assure inter-rater reliability. Moreover, the supervisor analyzed the coding by reviewers to reach consensus.

### c) Establishing construct validity & reliability

In the last part of phase 2, construct validity and reliability were established. The 4th version of the tool had 29 items divided under six constructs. It was an online google form comprising 3 parts; the first part was a brief description of the research questionnaire and its relevance to the residents along with informed consent. The second portion was about participant demographics and the third section was about the instrument. The items had to be marked on a 5-point Likert scale; Strongly Agree = 5, Agree = 4, Not sure = 3, Disagree = 2, Strongly disagree = 1. The form was piloted with 5 participants to verify efficient running.

#### Participants & procedure

The contributors were selected from the target population of postgraduate residents at various tertiary care hospitals undergoing FCPS-II training in various specialties of Medicine & Allied, Surgery & Allied, Gynecology and Pediatrics with more than six completed months of training.

There is no consensus in literature on a single best method to establish an appropriate sample size for factor analysis. Beavers proposed an item to participant ratio of 1:10 to calculate sample size provided the total variable number is greater than 19 [24]. Pilot testing and factor analysis in our study was done on a sample of 400 participants.

Google forms were sent to potential participants via email, shared in WhatsApp groups and also forwarded to supervisors to encourage trainees to respond. The initial instructions included an informed consent and an emphasis on maintaining confidentiality of data to encourage residents to give unbiased opinions.

#### Data analysis

There were 6 constructs and 29 items against these constructs, that we used to collect the data from 407 respondents (*n* = 407). Among all 29 items there were no non-response item against these six constructs. No item was used in reverse coding. During data entry in SPSS, since items were on the same Likert scale, they were reflected as continuous variables. Responses were categorized as “Strongly disagree, Disagree, Neither agree not disagree, Agree and Strongly agree” from 5 to 1 respectively.

The descriptive analyses were carried out through SPSS 26.0 while AMOS 25.0 was employed to conduct confirmatory factor analysis (CFA). Only confirmatory factor analysis (CFA) was carried out and exploratory factor analysis was not done as the factor structure was already found in previous studies [25]. Variance amongst the constructs were measured. Next, a model with 6 factors and 29 items was created. Factor loading was carried out using AMOS 25.0 and various model fit indices ( absolute fit, incremental fit and parsimonious fit indices) were employed to find out the appropriateness of the model [26] (Table 3).

Reliability was established through Cronbach Alpha and a value above 0.8 was considered as excellent while a value between 0.7 and 0.79 was considered acceptable [27].

## Results

### Phase I: Item development and qualitative content validation

The response rate was 100% and all experts gave their feedback. Items were revised, combined, and removed based on the feedback from 15 experts. Some items were re-worded to enhance clarity and reduce grammatical errors. Some items were shifted to another construct or merged. Roles and responsibilities was chosen as the first theme as only after knowing one’s roles and responsibilities one can go ahead with required collaboration. Double barrel questions were split which led to the addition of another item in the domain of collaborative leadership. This resulted in an instrument with a total of 30 items grouped under six themes (Annexure I- qualitative expert feedback).

### Phase II: Establishing validity & reliability of the instrument

#### Content validity index and content clarity average

About 25 experts with experience of working in multidisciplinary teams were emailed the second version of the questionnaire and asked to rate the statements for their relevance and clarity. Thirty items were assessed by seventeen experts according to their relevance in measuring the construct and clarity on 5- and 3-point Likert scales, respectively. All items’ I-CVIs were calculated. All the items had an I-CVI of more than 0.78 so none of the items were removed. Items with a CVI between 0.79 and 0.9 were reviewed and accepted after modification. The remaining items with I-CVI of more than 0.90 were accepted. Regarding clarity, 26 items had CCA > 2.4 and were accepted. Four items with CCA < 2.4 (T2S5, T3S1, T3S2, T3S5) were rephrased and accepted after amendments (Table 2). The average clarity of the scale was 2.8 and content validity index of scale (S-CVI) was 0.93 (Annexure II & III-CVI & CCA).

**Table 2:** I-CVI & CCA of individual items

S.No	Items	I-CVI	CCA	Decision
	**Theme 1: Roles/responsibilities for Intraprofessional collaborative practice**			
**T1S1**	I can share my learning with residents of other disciplines with ease when required	0.94	2.82	A
**T1S2**	I welcome the opportunity to work with other health professionals in small group learning activities for patient care	0.88	2.88	A
**T1S3**	I am aware of my role as well as my limitations in patient care	0.94	2.94	A
**T1S4**	I can approach other professionals in different disciplines for their particular expertise for multidisciplinary patient care	1.00	2.88	A
**T1S5**	I consistently give feedback to other residents in my setting about a relevant case when required	0.94	3	A
**T1S6**	I take feedback from my colleagues in other specialties positively	0.94	2.88	A
	**Theme 2: Intraprofessional collaborative communication**			
**T2S1**	My colleagues from other disciplines and I frequently communicate	0.88	2.88	A
**T2S2**	I understand when and what has to be communicated	1.00	2.88	A
**T2S3**	I can communicate timely according to the urgency of medical condition of patient.	0.94	2.82	A
**T2S4**	I am well versed with principles of written communication (should be timely, precise and in appropriate language)	0.88	2.94	A
**T2S5**	I discuss with other disciplines the degree to which each of us should be involved in a particular case	0.82	2.76	AM
	**Theme 3: Intraprofessional collaborative team-based care and networking**			
**T3S1**	I know many of the other residents personally	0.82	2.76	AM
**T3S2**	I know the workplace, resources and limitations of other specialties	0.88	2.88	AM
**T3S3**	I understand the referral and communication system	0.94	2.94	A
**T3S4**	I understand the perspective (viewpoint) of other disciplines’ residents in patient care	0.94	2.88	A
**T3S5**	I understand team dynamics and power relations	0.94	2.7	A
	**Theme 4: Values/ethics for intraprofessional collaborative practice**			
**T4S1**	I respect the roles, expertise and task distribution of other team members	1.00	2.94	A
**T4S2**	I respect the values of fellow residents related to patients’ outcome	0.94	2.88	A
**T4S3**	I am willing to cooperate with other residents without any preconceived notions	1.00	2.94	A
**T4S4**	I am not prejudiced against other specialties	0.82	2.88	AM
**T4S5**	I have the ability to look beyond my own position and task to get a wider picture	0.88	2.88	AM
**T4S6**	I work from a patient centered perspective in my practice	1.00	2.94	A
	**Theme 5: Sharing of mutual knowledge for IPCP**			
**T5S1**	I can willingly sacrifice a degree of autonomy (accept somebody else’s decision) to support cooperative problem solving	0.88	2.88	A
**T5S2**	I utilize formal and informal channels for problem-solving with my colleagues from other disciplines	0.82	2.82	AM
**T5S3**	Learning from other specialties gives a greater insight into the working of other disciplines	1.00	3	A
**T5S4**	I share mutual knowledge with other specialties to handle clinical cases effectively	1.00	2.94	A
	**Theme 6: Intraprofessional collaborative leadership**			
**T6S1**	I help my colleagues to address conflict with other disciplines effectively	1.00	2.94	A
**T6S2**	I can motivate and influence colleagues and build teams	0.92	3	A
**T6S3**	I can coordinate and plan collaborative meetings and processes	1.00	3	A
**T6S4**	I take responsibility of developing patient care plans when working in multidisciplinary teams	0.88	2.94	A
**T6S5**	I try to create a conducive environment where inputs from various specialties are encouraged and utilized towards patient centered care	1.00	2.94	A

I-CVI = Item CVI, A = Accepted, AM = Accepted after Modification, D = Deleted (Scale-CVI = 0.93)

### Response process validity

The instructions and verbal probing was understood by most of the respondents. Response process validity resulted in minor modifications in six items, rephrasing one item and deletion of one item (Annexure IV- Response Process Validity).

### Pilot testing the instrument

A total of 407 respondents (*n* = 407) contributed their responses. In the participants, 64% (262) were females while 36% (145) were males. The residents belonged to various specialties such as Medicine & Allied, Surgery & Allied, Pediatrics and Gynecology. The residents were in different phases of their training with 24% in the first year, 20% in second year, 15% in third year, 18% in fourth year and 23% in fifth year of training.

### Construct validity

**Fig. 2:**
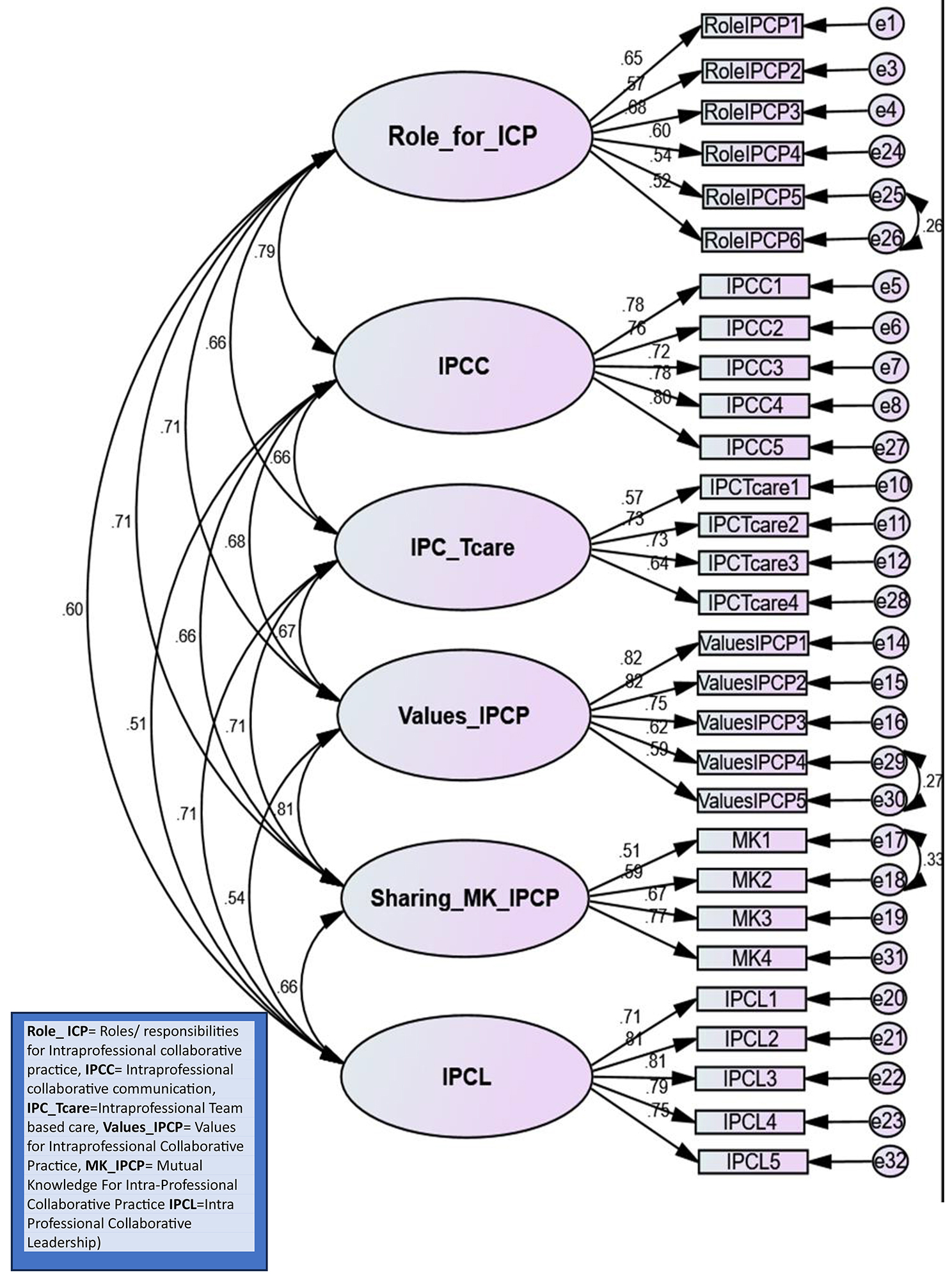
Sequential equation model for instrument

For CFA, the measurement model developed using AMOS 25.0 showed all the factors loaded with good regression weights and had strong mutual relationship. No item was removed as no item had a low loading value (i.e., < 0.50). Figure 2 displays the measurement model with factor loading. The model fit value of all indices were good so there was no need to delete any item for improving the goodness of fit. The Chi-Square value is less than 3 (i.e., 2.222), indicating excellent fit; P value less than or equal to 0.05 is highly significant (i.e., 0.000); indicating that the null hypothesis is accepted. In comparison to cutoff value, the Root Mean Square Error of Acceptance (RMSEA) value for the model is 0.055 that also shows an excellent model fit. According to cutoff values, the values of the comparison fit index (CFI) is slightly lower which is 0.922 which is acceptable, normal fit index (NFI), Tucker-Lewis index (TLI), and standard root mean residual (SRMR) are excellent indicating good model fit (Table 3).

**Table 3:** Recommended values-fit model indices

	Fit indices	Recommended cut-off values	Measured value
Incremental Fit Indices	Normed fit index	> 0.08 -1 0.09 is the cut-off value for a good fit	0.912
	Incremental fit index	Close to 1	0.92
	Relative fit index	Close to 1, the greater the better	0.851
Absolute Fit Indices	Root mean square error of approximation (RMSEA)	0.08–0.1 0.05 is the cut-off value for a good fit	0.055
	The goodness of fit index (GFI)	> 0.08-1 0.09- cut-off value for a good fit model	0.883
	Observed normed χ2 (CMIN/df)	< 5 The lesser the better	2.22
P value		> 0.05	0.059

### Reliability

Cronbach’s alpha of the instrument was 0.937. There were a total of six latent variables in this instrument: Roles & responsibilities, Intraprofessional collaborative communication, Intraprofessional collaborative team-based care, values & ethics for IPCP, Sharing of mutual knowledge for IPCP and Intraprofessional collaborative leadership and Cronbach’s alpha values were 0.773, 0.877, 0.751, 0.844, 0.749 and 0.881 respectively. It showed that all the variables in this study operationalized satisfactorily. Moreover, the corrected item-to-total correlation (CITC) for most of the items was > 0.2, (Annexure V) which showed that each item fitted in its respective subscale [28].

**Fig. 3:**
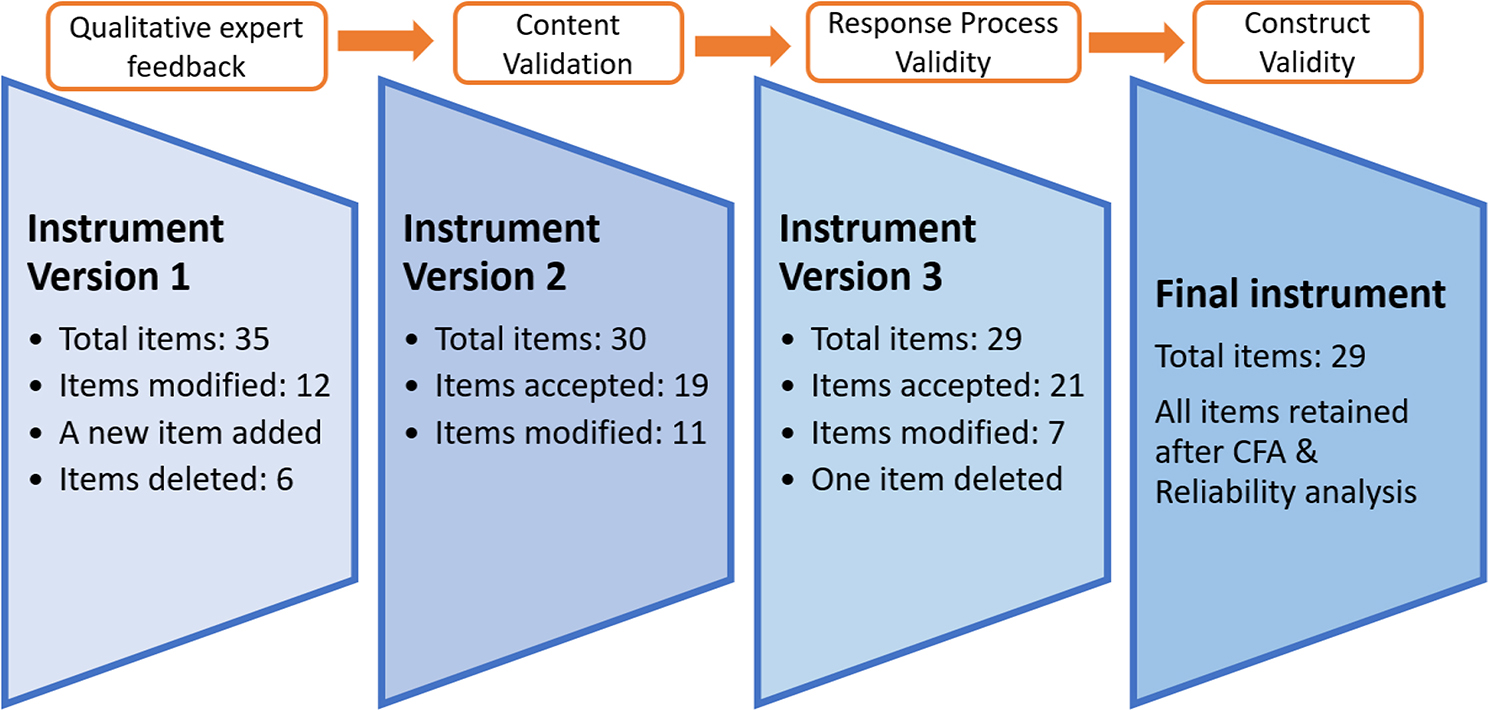
Changes during the evolution of the instrument (IPCP-R)

The final version of the tool had 29 items grouped under six themes (Annexure VI). The changes in the instrument at various stages are summarized in Fig. 3.

## Discussion

Considering the need of new strategies required to incorporate and assess the multi-dimensional competency of collaboration in residents and a lack of validated assessment tools, the present mixed-method study aimed at development and validation a self-assessment instrument that residents of various specialties can use to self-assess their intraprofessional collaborative practices while working in multi-disciplinary environment in hospitals.

Collaboration in healthcare is a topic that has aroused much interest over the years and a number of instruments have been developed for its assessment. However, the instruments vary in the construct being measured. Some of the research evaluated people’s attitudes, behaviors, and personal opinions, such as how doctors and specialists rated collaboration [29], measuring perceptions of collaboration [30] and nurse-physician judgments of intraprofessional collaboration [31]. Other studies validated instruments to assess collaborative relationships [32], measuring IPC between clinical practitioners at various tiers of care, evaluating team function [33] and internal participation (a central part of patient-centered teamwork) in both healthcare professionals and patients.

The instruments developed to evaluate collaboration measured collaboration against various constructs. The key constructs of the Modified Index of Interdisciplinary Collaboration (MIIC) are interdependence in roles, joint ownership of objectives, reflection on progression, newly created professional undertakings and professional flexibility as its key constructs. The Assessment of Interprofessional Team Collaboration Scale (AITCS) had items grouped under the themes of partnerships, coordination and cooperation. The factor structure of Doctors Opinion on Collaboration (DOC) scale included knowing each other, communication, organization, professional expertise and image. The Collaborative Practice Assessment tool (CPAT), [34] tool identified roles and responsibilities, a meaningful purpose, communication, conflict management and coordination of care, team leaderships and relationships along with a patient perspective as its key component themes. T-MEX identified networking as supportive team relationships [35]. These themes are overall similar to the ones identified in our study.

A sample of nurses, clinicians, and administrators in the hospice context were used to examine the validity, reliability, and interpretability of the MIIC, a modified version of the IIC [30]. Five hospice workers established the content validity, and no factor analysis was performed. Orchard et al. (2012) developed the (AITCS) to evaluate collaborative interactions in healthcare using Sullivan’s concept of collaboration as a guide. A 47-item scale was the end result, and it was refined further using confirmatory and exploratory factor analysis. However, the study’s sample size was modest. The Collaborative Practice Assessment Tool (CPAT) identified relative strengths and shortcomings of teams in hospice settings [34]. The first phase’s items were created using literature research and professional judgment. EFA was used to improve the questionnaire, but the sample size was too small to determine precise structural validity.

These previous studies followed a mixed method approach of both qualitative and quantitative validation similar to what was used for our project. They were based on the theoretical models of collaboration that we also reviewed and incorporated in our study, their target population had a very wide and different range incorporating pharmacists, nurses and even patients at times so it was difficult to find measurement equivalence in such a large diversity of professionals. In contrast our instrument focusses solely on collaboration amongst residents in multidisciplinary environments and is more targeted in this regard. The residents can use it to self-assess and reflect on their intraprofessional collaborative practices.

In previous tools on interprofessional collaboration studied at various levels, items were developed through literature review, as well as focus group discussions and latent factors and domains were extracted through EFA [29, 30]. The items of our instrument were developed using multiple competency frameworks as well as systematic reviews of previously developed tools for assessment of collaboration in healthcare settings [10, 36] and [37]. Thus, the instrument covers all aspects of intraprofessional collaborative practices in residents to provide a holistic and comprehensive self-evaluation.

For instrument validation, researchers take help from qualitative expert validation and further strengthening of the instrument is done from quantitative estimation through CVI [38]. Oliver et al. did qualitative content validation [30]and the AITCS developed by Orchard et al., also relied on qualitative feedback from interprofessional collaboration experts [32]. We performed qualitative feedback followed by calculation of CVI and CCA. I-CVI and S-CVI of our instrument were well above the desired range.

Response process validity of the respondents on the survey items determined through cognitive pre-testing of the statements was found to be very beneficial in the modification of several items based on comprehension of the participants. None of the other scales on teamwork or interprofessional collaboration or interprofessional relationships has checked this validity.

The previous scales such as the DOC as well as MIIC used EFA to establish construct validity. In our study, only CFA was done due to certain expectations regarding the number of factors and their correlations and the specific items that reflected the factors [26, 39]. The model developed in CFA established satisfactory construct validity (c^2^ = 2.22). An overall good fit of the 6-factor model was demonstrated by the values of the absolute and incremental fit indices.

The final instrument composed of 29 items had good content validity and response process validity. The results of our study are promising as the developed model with 29 items showed a good fit with values of Chi-Square and other fit indices within good range.

The study demonstrated an acceptable level of internal consistency with Cronbach’s α for the total scale of 0.93 with values for subscales ranging from 0.75 to 0.88. Another good measure of internal consistency is Corrected item-to-total correlation (CITC). CITC for most of the items in the subscales of the instrument was > 0.2, which showed that the items belonged to the subscale [19]. The DOC, MIIC and AITCS all had a Cronbach alpha value of greater than 0.9.

The study has several limitations. Firstly, our experts feedback panel comprised only national experts. The experts included had vast experience of working in multidisciplinary teams and were suitable for the panel; they understood the problems faced while working in such an environment. Also, most of the experts were supervisors and worked closely with residents and understood how residents work. Secondly, it’s a self-assessment tool, therefore it is prone to respondent’s bias and lack of observation. Another limiting factor was that the majority of data for factor analysis was collected mainly from government hospitals and private hospitals were not represented. The study also did not include dentists and residents of non-clinical specialties such as radiology and pathology which are also part of multidisciplinary decision-making. For pilot testing of instrument, purposive convenience sampling was done instead of random sampling. Thus, results may not be generalized to entire population of post-graduate residents.

Postgraduate residents need to be aware of their central role in patient care and be mindful of effective collaboration for efficient and safe patient outcomes. We recommend using this self-assessment tool to identify collaborative practices in residents and its utility to identify any lapses in conduct or attitude leading to their inability to work productively in multidisciplinary teams. This tool can be used by residents of all major specialties to assess themselves and reflect on their collaborative practices and behavior. Since effective collaboration produces good results for the organization, this tool can be added to resident portfolios for periodic evaluations. Moreover, courses about collaborative practices need to be developed and administered to residents and this tool can be used to assess them (pre-test & post-test).

The research also opens up further avenues for potential investigation into the validity of the instrument in other contexts and countries. It should be validated on larger random samples from different professional groups including dentists and non-clinical specialty residents and in different settings countrywide and globally. We also suggest further research to design and implement courses incorporating intraprofessional collaboration in residents at postgraduate level to train future consultants in the norms of working in a healthy collaborative environment.

## Conclusion

The findings of this study suggest that the IPCP-R has an appropriate level of content, measures intra-professional collaboration reliably and is representative of the focus population i.e., postgraduate residents. The scale measures six domains of intraprofessional collaboration covering most aspects of intraprofessional collaboration in healthcare. Team-based care and networking, values and ethics, sharing of mutual knowledge and collaborative leadership emerged as the key constructs for the instrument.

The original version of the tool underwent substantial changes during the validation process and final version composed of 29 items was found reliable and valid by psychometric analysis.

Considering the nonexistence of tools to assess collaborative practices in postgraduate residents working in multidisciplinary environments, this tool is an important advancement in terms of analyzing and reporting collaborative practices and the factors leading to a lack of it. This will help propose solutions to address these issues with an aim to enhance positive teamwork in a collaborative environment for efficient and safe patient care.

### Electronic supplementary material

Below is the link to the electronic supplementary material.

Supplementary Material 1

## Data Availability

The datasets used or analyzed during the current study are available from the corresponding author on reasonable request.

## Abbreviations

AMEE: Association for American Association Europe
AITCS: Assessment of Interprofessional Team Collaboration Scale
CCA: Content Clarity Average
CFA: Confirmatory Factor Analysis
CITC: Corrected Item Total Correlation
DOC: Doctors Opinion on Collaboration
EFA: Exploratory Factor Analysis
CPAT: Collaborative Practice Assessment Tool
CVI: Content Validity Index
DOC: Doctors Opinion on Collaboration
EFA: Exploratory Factor Analysis
IPC: Inter Professional Collaboration
IPCP: Intra Professional Collaborative Practices
IPCP-R: Intra Professional Collaborative Practices in Residents
T-MEX: Teamwork Mini Evaluation Exercise
