# Supplementary material for: Collaboration cognizance: Development of a self-assessment tool to measure intra-professional collaborative practices (IPCP) in postgraduate medical residents at tertiary care hospitals
Source: BMC Med Educ. 2024 Jul 19;24:771. doi: 10.1186/s12909-024-05759-7 (PMC11264871; doi:10.1186/s12909-024-05759-7)
Supplement: Supplementary file 1 — Supplementary Material 1 [file 12909_2024_5759_MOESM1_ESM.docx]

Annexure I: Table of Expert Feedback for Qualitative Content Validation

| **S No** | **Items** | **Accept** | **Modify** | **Delete (Please give reason)** | **New item suggested/ comments** |
| --- | --- | --- | --- | --- | --- |
| **Theme 1: Intraprofessional collaborative communication** | | | | | |
| T1S1 | My colleagues from other disciplines and I frequently communicate | Accept |  |  |  |
| T1S2 | I understand when and what has to be communicated | Accept |  |  |  |
| T1S3 | I can communicate timely according to the urgency of medical condition of patient. | Accept |  |  |  |
| T1S4 | I am well versed with principles of written communication |  | Modify |  | Mention principles of written communication for clarity (SS & NA) Accepted |
| T1S5 | I discuss with other disciplines the degree to which each of us should be involved in a particular case | Accept |  |  |  |
| **Theme 2: Roles/responsibilities for Intraprofessional collaborative practice** | | | | | |
| T2S1 | I can share my learning with residents of other profession with ease when required |  | Move theme to first place as roles come before communication (Accepted) FS |  | Instead of other profession, use the word other disciplines (Accepted) FS, SS, AR  Share knowledge with other disciplines (KQ) Accepted |
| T2S2 | I welcome the opportunity to work with other health professionals in small group projects |  | Modify |  | Small group learning activities for patient care (KQ) Accepted |
| T2S3 | I am aware of my role as well as my limitations in patient care | Accept |  |  |  |
| T2S4 | I can utilize other professionals in different disciplines for their particular expertise |  | Modify |  | I can utilize other professionals in different disciplines for a multidisciplinary patient approach (NA)  I can approach (SN) Accepted |
| T2S5 | I consistently give feedback to other residents in my setting about a relevant case |  | Modify |  | As and when required (SN) Accepted |
| T2S6 | I take feedback from my colleagues in other specialties positively | Accept |  |  |  |
| **Theme 3: Intraprofessional collaborative team-based care and networking** | | | | | |
| T3S1 | I know many of the other residents personally | Accept |  |  |  |
| T3S2 | I know the workplace, resources and limitations of other specialties | Accept |  |  |  |
| T3S3 | I know the referral and communication system |  | Modify |  | Repetition of communication (KQ)  I understand.. and its limitations (SN) Accepted |
| T3S4 | I understand the profession and perspective of other residents and come up to expectations |  | Modify |  | Change the word perspective (simplify) (KQ) Accepted  Simplify (SN) Accepted  Remove expectations to clarify (SN) |
| T3S4 | I understand team dynamics and power relations |  | Modify |  | Team and power dynamics, need to rephrase (KQ) |
| **Theme 4: Values/ethics for intraprofessional collaborative practice** | | | | | |
| T4S1 | I respect each other’s roles, expertise and task distribution. |  | Modify |  | Who are each other? Specify (KQ) Accepted  I accept .. of other team members (SN) accepted |
| T4S2 | I respect each other’s values related to the patients’ outcome |  | Modify |  | Values of other team members (SN) Accepted |
| T4S3 | I am willing to cooperate with an open mind |  | Modify |  | Without preconceived notions (SN) Accepted |
| T4S4 | I am not prejudiced against other specialties | Accept |  |  | Disciplines (SS) accepted |
| T4S5 | I have the ability to look beyond my own position and task |  | Modify |  | Elaborate (to get a wider picture) (AK) Accepted |
| T4S6 | I work from a patient centered perspective in my practice | Accept |  |  |  |
| **Theme 5: Sharing of mutual knowledge for IPCP** | | | | | |
| T5S1 | I can willingly sacrifice a degree of autonomy to support cooperative problem solving. |  | Modify |  | I can willingly accept somebody else’s decision/advice (FS) |
| T5S2 | I utilize formal and informal procedures for problem-solving with my colleagues from other disciplines |  |  |  | Replace procedures with channels (FS)  Rephrase (AR) |
| T5S3 | I learn from residents of other specialties, and it helps overcome stereotypes about other specialties |  | Modify |  | Learning from other specialties gives a greater insight (FS) Accepted  Rephrase stereotype (SN)  Repetition, same as T2S1, (KQ) |
| T5S4 | I share mutual knowledge with other specialties to handle clinical cases effectively | Accept |  |  |  |
| **Theme 6: Intraprofessional collaborative leadership** | | | | | |
| T6S1 | I help my colleagues to address conflict with other professionals directly. |  | Modify |  | I help my colleagues address conflict with other disciplines effectively (FS) |
| T6S2 | I can motivate and influence colleagues and build teams | Accept |  |  |  |
| T6S3 | I can coordinate and plan collaborative meetings and processes | Accept |  |  |  |
| T6S4 | I take responsibility of developing treatment plans of patient when working in multidisciplinary teams |  |  |  | Patient care plans (SN, AC) Accepted |

A new statement proposed in the domain of leadership: T6S5 I try to create a conducive environment where inputs from various specialties are encouraged and utilized towards patient centered care

Annexure II: Calculation of I-CVI for Content Validation

| Sr No | Items | E1 (KQ) | E2 (SN) | E3 (AD) | E4 (AA) | E5 (AK) | E6 (Sn) | E7 (SS) | E8 (GA) | E9 (FS) | E10 (I) | E11 (AR) | E12 (AC) | E13 (TA) | E14 (SJ) | E15 (AA) | E16-R | E17-R | I-CVI | Decision |  |
| --- | --- | --- | --- | --- | --- | --- | --- | --- | --- | --- | --- | --- | --- | --- | --- | --- | --- | --- | --- | --- | --- |
|  | **Theme 1: Roles/responsibilities for Intraprofessional collaborative practice** | | | | | | | | | | | | | | | | | | | | |
| T1S1 | I can share my learning with residents of other disciplines with ease when required | QR  1 | QR  1 | VR  1 | VR  1 | VR  1 | VR  1 | VR  1 | VR  1 | QR  1 | QR  1 | S  R  0 | QR  1 | Q  R  1 | VR  1 | VR  1 | VR  1 | VR  1 | 0.94 | A |  |
| T1S2 | I welcome the opportunity to work with other health professionals in small group learning activities for patient care | V R  1 | VR  1 | VR  1 | VR  1 | VR  1 | VR  1 | VR  1 | VR  1 | S  R  0 | VR  1 | QR  1 | VR  1 | VR  1 | VR  1 | VR  1 | S  R  0 | VR  1 | 0.88 | A |  |
| T1S3 | I am aware of my role as well as my limitations in patient care | VR  1 | VR  1 | VR  1 | VR  1 | QR  1 | VR  1 | VR  1 | VR  1 | VR  1 | VR  1 | QR  1 | VR  1 | VR  1 | VR  1 | VR  1 | VR  1 | S  R  0 | 0.94 | A |  |
| T1S4 | I can approach other professionals in different disciplines for their particular expertise for multidisciplinary patient care | VR  1 | VR  1 | VR  1 | VR  1 | QR  1 | VR  1 | VR  1 | VR  1 | QR  1 | QR  1 | QR  1 | VR  1 | VR  1 | VR  1 | VR  1 | QR  1 | VR  1 | 1.00 | A |  |
| T1S5 | I consistently give feedback to other residents in my setting about a relevant case when required | VR  1 | QR  1 | VR  1 | V  R  1 | VR  1 | VR  1 | VR  1 | QR  1 | VR  1 | S  R  0 | VR  1 | VR  1 | V  R  1 | V  R  1 | Q  R  1 | Q  R  1 | Q  R  1 | 0.94 | A |  |
| T1S6 | I take feedback from my colleagues in other specialties positively | VR  1 | QR  1 | VR  1 | VR  1 | VR  1 | QR  1 | VR  1 | QR  1 | VR  1 | V  R  1 | S  R  0 | VR  1 | VR  1 | Q  R  1 | V  R  1 | V  R  1 | VR  1 | 0.94 | A |  |
|  | **Theme 2: Intraprofessional collaborative communication** | | | | | | | | | | | | | | | | | | | |  |
| T2S1 | My colleagues from other disciplines and I frequently communicate | QR  1 | VR  1 | VR  1 | VR  1 | VR  1 | VR  1 | VR  1 | VR  1 | QR  1 | VR  1 | S  R  0 | QR  1 | VR  1 | VR  1 | S  R  0 | QR  1 | VR  1 | 0.88 | A |  |
| T2S2 | I understand when and what has to be communicated | QR  1 | VR  1 | VR  1 | VR  1 | VR  1 | QR  1 | VR  1 | QR  1 | QR  1 | QR  1 | QR  1 | QR  1 | QR  1 | VR  1 | VR  1 | VR  1 | QR  1 | 1.00 | A |  |
| T2S3 | I can communicate timely according to the urgency of medical condition of patient. | VR  1 | VR  1 | VR  1 | VR  1 | VR  1 | VR  1 | VR  1 | VR  1 | QR  1 | VR  1 | QR  1 | VR  1 | VR  1 | VR  1 | S  R  0 | VR  1 | VR  1 | 0.94 | A |  |
| T2S4 | I am well versed with principles of written communication (should be timely, precise and in appropriate language) | VR  1 | VR  1 | VR  1 | VR  1 | VR  1 | VR  1 | VR  1 | VR  1 | S  R  0 | VR  1 | QR  1 | VR  1 | VR  1 | VR  1 | VR  1 | QR  1 | S  R  0 | 0.88 | A |  |
| T2S5 | I discuss with other disciplines the degree to which each of us should be involved in a particular case | S  R  0 | VR  1 | VR  1 | S  R  0 | QR  1 | S  R  0 | VR  1 | VR  1 | Q  R  1 | Q  R  1 | QR  1 | V  R  1 | V  R  1 | VR  1 | VR  1 | VR  1 | VR  1 | 0.82 | A  M |  |
|  | **Theme 3: Intraprofessional collaborative team-based care and networking** | | | | | | | | | | | | | | | | | | | |  |
| T3S1 | I know many of the other residents personally | VR  1 | VR  1 | VR  1 | VR  1 | VR  1 | NR  0 | VR  1 | QR  1 | QR  1 | S  R  0 | S  R  0 | VR  1 | VR  1 | QR  1 | S  R  0 | VR  1 | VR  1 | 0.82 | A  M |  |
| T3S2 | I know the workplace, resources and limitations of other specialties | VR  1 | VR  1 | VR  1 | VR  1 | VR  1 | S  R  0 | VR  1 | QR  1 | VR  1 | QR  1 | QR  1 | VR  1 | QR  1 | QR  1 | QR  1 | S  R  0 | VR  1 | 0.88 | A  M |  |
| T3S3 | I understand the referral and communication system | VR  1 | VR  1 | VR  1 | VR  1 | VR  1 | VR  1 | VR  1 | VR  1 | VR  1 | VR  1 | QR  1 | VR  1 | S  R  0 | V  R  1 | VR  1 | V  R  1 | VR  1 | 0.94 | A |  |
| T3S4 | I understand the perspective (viewpoint) of other disciplines’ residents in patient care | VR  1 | VR  1 | VR  1 | VR  1 | VR  1 | VR  1 | VR  1 | QR  1 | VR  1 | QR  1 | QR  1 | VR  1 | VR  1 | VR  1 | S  R  0 | V  R  1 | VR  1 | 0.94 | A |  |
| T3S5 | I understand team dynamics and power relations | VR  1 | VR  1 | VR  1 | QR  1 | VR  1 | VR  1 | VR  1 | QR  1 | N  R  0 | VR  1 | QR  1 | VR  1 | VR  1 | VR  1 | VR  1 | VR  1 | QR  1 | 0.94 | A |  |
|  | **Theme 4: Values/ethics for intraprofessional collaborative practice** | | | | | | | | | | | | | | | | | | | |  |
| T4S1 | I respect the roles, expertise and task distribution of other team members | VR  1 | VR  1 | VR  1 | VR  1 | VR  1 | VR  1 | VR  1 | VR  1 | V  R  1 | VR  1 | QR  1 | VR  1 | VR  1 | VR  1 | QR  1 | VR  1 | VR  1 | 1.00 | A |  |
| T4S2 | I respect the values of fellow residents related to patients’ outcome | VR  1 | VR  1 | VR  1 | S  R  0 | VR  1 | QR  1 | VR  1 | VR  1 | QR  1 | VR  1 | QR  1 | VR  1 | VR  1 | VR  1 | VR  1 | QR  1 | QR  1 | 0.94 | A |  |
| T4S3 | I am willing to cooperate with other residents without any preconceived notions | VR  1 | VR  1 | VR  1 | VR  1 | VR  1 | VR  1 | VR  1 | VR  1 | QR  1 | QR  1 | QR  1 | VR  1 | VR  1 | VR  1 | VR  1 | QR  1 | QR  1 | 1.00 | A |  |
| T4S4 | I am not prejudiced against other specialties | VR  1 | VR  1 | VR  1 | VR  1 | QR  1 | NR  0 | VR  1 | VR  1 | QR  1 | VR  1 | S  R  0 | VR  1 | VR  1 | VR  1 | VR  1 | QR  1 | S  R  0 | 0.82 | A  M |  |
| T4S5 | I have the ability to look beyond my own position and task to get a wider picture | S  R  0 | VR  1 | VR  1 | VR  1 | QR  1 | S  R  0 | VR  1 | VR  1 | QR  1 | QR  1 | QR  1 | S  R  0 | QR  1 | V  R  1 | V  R  1 | QR  1 | QR  1 | 0.88 | A  M |  |
| T4S6 | I work from a patient centered perspective in my practice | QR  1 | VR  1 | VR  1 | VR  1 | VR  1 | QR  1 | VR  1 | QR  1 | VR  1 | VR  1 | QR  1 | V  R  1 | VR  1 | VR  1 | VR  1 | VR  1 | VR  1 | 1.00 | A |  |
|  | **Theme 5: Sharing of mutual knowledge for IPCP** | | | | | | | | | | | | | | | | | | | |  |
| T5S1 | I can willingly sacrifice a degree of autonomy (accept somebody else’s decision) to support cooperative problem solving | S  R  0 | VR  1 | VR  1 | VR  1 | QR  1 | VR  1 | VR  1 | QR  1 | VR  1 | VR  1 | QR  1 | S  R  0 | VR  1 | VR  1 | VR  1 | VR  1 | QR  1 | 0.88 | A |  |
| T5S2 | I utilize formal and informal channels for problem-solving with my colleagues from other disciplines | S  R  0 | QR  1 | VR  1 | VR  1 | QR  1 | QR  1 | VR  1 | QR  1 | QR  1 | VR  1 | S  R  0 | S  R  0 | VR  1 | QR  1 | QR  1 | QR  1 | QR  1 | 0.82 | A  M |  |
| T5S3 | Learning from other specialties gives a greater insight into the working of other disciplines | QR  1 | QR  1 | VR  1 | VR  1 | QR  1 | VR  1 | VR  1 | VR  1 | VR  1 | VR  1 | QR  1 | QR  1 | QR  1 | QR  1 | VR  1 | VR  1 | VR  1 | 1.00 | A |  |
| T5S4 | I share mutual knowledge with other specialties to handle clinical cases effectively | QR  1 | VR  1 | VR  1 | VR  1 | VR  1 | VR  1 | VR  1 | VR  1 | VR  1 | VR  1 | QR  1 | QR  1 | VR  1 | VR  1 | VR  1 | VR  1 | QR  1 | 1.00 | A |  |
|  | **Theme 6: Intraprofessional collaborative leadership** | | | | | | | | | | | | | | | | | | | |  |
| T6S1 | I help my colleagues to address conflict with other disciplines effectively | QR  1 | VR  1 | VR  1 | VR  1 | VR  1 | S  R  1 | VR  1 | VR  1 | VR  1 | VR  1 | QR  1 | Q  R  1 | VR  1 | VR  1 | VR  1 | VR  1 | VR  1 | 1.00 | A |  |
| T6S2 | I can motivate and influence colleagues and build teams | QR  1 | S  R  0 | VR  1 | VR  1 | VR  1 | Q  R  1 | VR  1 | VR  1 | VR  1 | VR  1 | QR  1 | QR  1 | QR  1 | QR  1 | QR  1 | QR  1 | QR  1 | 0.92 | A |  |
| T6S3 | I can coordinate and plan collaborative meetings and processes | QR  1 | VR  1 | VR  1 | VR  1 | VR  1 | QR  1 | VR  1 | QR  1 | VR  1 | VR  1 | QR  1 | Q  R  1 | VR  1 | VR  1 | VR  1 | VR  1 | QR  1 | 1.00 | A |  |
| T6S4 | I take responsibility of developing patient care plans when working in multidisciplinary teams | S  R  0 | VR  1 | VR  1 | VR  1 | VR  1 | VR  1 | VR  1 | VR  1 | VR  1 | VR  1 | QR  1 | S  R  0 | VR  1 | VR  1 | QR  1 | QR  1 | QR  1 | 0.88 | A |  |
| T6S5 | I try to create a conducive environment where inputs from various specialties are encouraged and utilized towards patient centered care | QR  1 | VR  1 | VR  1 | VR  1 | QR  1 | VR  1 | VR  1 | VR  1 | VR  1 | VR  1 | QR  1 | Q  R  1 | V  R  1 | VR  1 | VR  1 | VR  1 | VR  1 | 1.00 | A |  |

VR=Very Relevant (1) I-CVI= Content validity index of items

QR=Quite Relevant (1) A= Accepted

SR=Needs slight revisions to be relevant (0) AM= Accepted after Modification

NR=Not Relevant (0) D= Deleted

Scale-CVI=92.96

Annexure III: Table of Content Clarity Average (CCA) after Expert Validation

| Sr No | Items | E1 (KQ) | E2 (SN) | E3 (AD) | E4 (AA) | E5 (AK) | E6 (Sn) | E7 (SS) | E8 (GA) | E9 (FS) | E10 (I) | E11 AR) | E12(AC) | E13(TA) | E14(SJ) | E15(AA) | E16-R | E17-R | CCA | Decision |
| --- | --- | --- | --- | --- | --- | --- | --- | --- | --- | --- | --- | --- | --- | --- | --- | --- | --- | --- | --- | --- |
|  | **Theme 1: Roles/responsibilities for Intraprofessional collaborative practice** | | | | | | | | | | | | | | | | | | | |
| T1S1 | I can share my learning with residents of other disciplines with ease when required | VC  3 | VC  3 | VC  3 | VC  3 | VC  3 | VC  3 | VC  3 | VC  3 | NC  1 | VC  3 | R  2 | VC  3 | VC  3 | VC  3 | VC  3 | VC  3 | VC  3 | 2.82 | A |
| T1S2 | I welcome the opportunity to work with other health professionals in small group learning activities for patient care | V C  3 | VC  3 | VC  3 | VC  3 | VC  3 | VC  3 | VC  3 | VC  3 | NC  1 | VC  3 | VC  3 | VC  3 | VC  3 | VC  3 | VC  3 | VC  3 | VC  3 | 2.88 | A |
| T1S3 | I am aware of my role as well as my limitations in patient care | VC  3 | VC  3 | VC  3 | VC  3 | VC  3 | VC  3 | VC  3 | VC  3 | VC  3 | VC  3 | VC  3 | R  2 | VC  3 | VC  3 | VC  3 | VC  3 | VC  3 | 2.94 | A |
| T1S4 | I can approach other professionals in different disciplines for their particular expertise for multidisciplinary patient care | VC  3 | VC  3 | VC  3 | VC  3 | VC  3 | VC  3 | VC  3 | VC  3 | R  2 | VC  3 | VC  3 | R  2 | VC  3 | VC  3 | VC  3 | VC  3 | VC  3 | 2.88 | A |
| T1S5 | I consistently give feedback to other residents in my setting about a relevant case when required | VC  3 | VC  3 | VC  3 | VC  3 | VC  3 | VC  3 | VC  3 | VC  3 | VC  3 | VC  3 | V  C  3 | VC  3 | VC  3 | VC  3 | VC  3 | VC  3 | VC  3 | 3 | A |
| T1S6 | I take feedback from my colleagues in other specialties positively | VC  3 | VC  3 | VC  3 | VC  3 | VC  3 | VC  3 | VC  3 | VC  3 | R  2 | VC  3 | R  2 | V  C  3 | VC  3 | VC  3 | VC  3 | VC  3 | VC  3 | 2.88 | A |
|  | **Theme 2: Intraprofessional collaborative communication** | | | | | | | | | | | | | | | | | | | |
| T2S1 | My colleagues from other disciplines and I frequently communicate | VC  3 | VC  3 | VC  3 | R  2 | VC  3 | VC  3 | VC  3 | VC  3 | VC  3 | VC  3 | R  2 | VC  3 | VC  3 | VC  3 | VC  3 | VC  3 | VC  3 | 2.88 | A |
| T2S2 | I understand when and what has to be communicated | VC  3 | VC  3 | VC  3 | VC  3 | VC  3 | VC  3 | VC  3 | VC  3 | NC  1 | VC  3 | VC  3 | VC  3 | VC  3 | VC  3 | VC  3 | VC  3 | VC  3 | 2.88 | A |
| T2S3 | I can communicate timely according to the urgency of medical condition of patient. | VC  3 | VC  3 | VC  3 | VC  3 | VC  3 | VC  3 | VC  3 | VC  3 | NC  1 | VC  3 | VC  3 | VC  3 | R  2 | VC  3 | VC  3 | VC  3 | VC  3 | 2.82 | A |
| T2S4 | I am well versed with principles of written communication (should be timely, precise and in appropriate language) | VC  3 | VC  3 | VC  3 | VC  3 | VC  3 | VC  3 | VC  3 | VC  3 | R  2 | VC  3 | VC  3 | VC  3 | VC  3 | VC  3 | VC  3 | VC  3 | VC  3 | 2.94 | A |
| T2S5 | I discuss with other disciplines the degree to which each of us should be involved in a particular case | R  2 | VC  3 | VC  3 | VC  3 | VC  3 | R  2 | VC  3 | VC  3 | NC  1 | VC  3 | VC  3 | VC  3 | VC  3 | VC  3 | VC  3 | VC  3 | VC  3 | 2.76 | A  M |
|  | **Theme 3: Intraprofessional collaborative team-based care and networking** | | | | | | | | | | | | | | | | | | | |
| T3S1 | I know many of the other residents personally | VC  3 | VC  3 | VC  3 | VC  3 | VC  3 | V  C  3 | VC  3 | VC  3 | VC  1 | VC  3 | R  2 | VC  3 | R  2 | VC  3 | VC  3 | VC  3 | VC  3 | 2.76 | A  M |
| T3S2 | I know the workplace, resources and limitations of other specialties | VC  3 | VC  3 | VC  3 | VC  3 | VC  3 | VC  3 | VC  3 | VC  3 | R  2 | VC  3 | VC  3 | VC  3 | VC  3 | R  2 | VC  3 | VC  3 | VC  3 | 2.88 | A  M |
| T3S3 | I understand the referral and communication system | VC  3 | VC  3 | VC  3 | VC  3 | VC  3 | VC  3 | VC  3 | VC  3 | VC  3 | VC  3 | VC  3 | VC  3 | R  2 | VC  3 | VC  3 | VC  3 | VC  3 | 2.94 | A |
| T3S4 | I understand the perspective (viewpoint) of other disciplines’ residents in patient care | VC  3 | VC  3 | VC  3 | VC  3 | VC  3 | VC  3 | VC  3 | VC  3 | R  2 | VC  3 | VC  3 | VC  3 | VC  3 | VC  3 | VC  3 | VC  3 | R  2 | 2.88 | A |
| T3S5 | I understand team dynamics and power relations | VC  3 | VC  3 | VC  3 | R  2 | VC  3 | VC  3 | VC  3 | VC  3 | NC  1 | VC  3 | VC  3 | VC  3 | VC  3 | VC  3 | VC  3 | R  2 | R  2 | 2.7 | A  M |
|  | **Theme 4: Values/ethics for intraprofessional collaborative practice** | | | | | | | | | | | | | | | | | | | |
| T4S1 | I respect the roles, expertise and task distribution of other team members | VC  3 | VC  3 | VC  3 | VC  3 | VC  3 | VC  3 | VC  3 | VC  3 | VC  3 | VC  3 | VC  3 | VC  3 | VC  3 | VC  3 | VC  3 | R  2 | VC  3 | 2.94 | A |
| T4S2 | I respect the values of fellow residents related to patients’ outcome | VC  3 | VC  3 | VC  3 | NC  1 | VC  3 | VC  3 | VC  3 | VC  3 | R  2 | VC  3 | VC  3 | VC  3 | R  2 | VC  3 | VC  3 | VC  3 | VC  3 | 2.88 | A |
| T4S3 | I am willing to cooperate with other residents without any preconceived notions | VC  3 | VC  3 | VC  3 | VC  3 | VC  3 | VC  3 | VC  3 | VC  3 | R  2 | VC  3 | VC  3 | VC  3 | VC  3 | VC  3 | VC  3 | VC  3 | VC  3 | 2.94 | A |
| T4S4 | I am not prejudiced against other specialties | VC  3 | VC  3 | VC  3 | VC  3 | VC  3 | VC  3 | VC  3 | VC  3 | R  2 | VC  3 | R  2 | VC  3 | VC  3 | VC  3 | VC  3 | VC  3 | VC  3 | 2.88 | A |
| T4S5 | I have the ability to look beyond my own position and task to get a wider picture | R  2 | VC  3 | VC  3 | VC  3 | VC  3 | VC  3 | VC  3 | VC  3 | R  2 | VC  3 | VC  3 | VC  3 | VC  3 | VC  3 | VC  3 | VC  3 | VC  3 | 2.88 | A |
| T4S6 | I work from a patient centered perspective in my practice | VC  3 | VC  3 | VC  3 | VC  3 | VC  3 | R  2 | VC  3 | VC  3 | VC  3 | VC  3 | VC  3 | VC  3 | VC  3 | VC  3 | VC  3 | VC  3 | VC  3 | 2.94 | A |
|  | **Theme 5: Sharing of mutual knowledge for IPCP** | | | | | | | | | | | | | | | | | | | |
| T5S1 | I can willingly sacrifice a degree of autonomy (accept somebody else’s decision) to support cooperative problem solving | R  2 | VC  3 | VC  3 | VC  3 | VC  3 | VC  3 | VC  3 | VC  3 | R  2 | VC  3 | VC  3 | VC  3 | VC  3 | VC  3 | VC  3 | VC  3 | VC  3 | 2.88 | A |
| T5S2 | I utilize formal and informal channels for problem-solving with my colleagues from other disciplines | R  2 | VC  3 | VC  3 | VC  3 | VC  3 | VC  3 | VC  3 | VC  3 | R  2 | VC  3 | R  2 | VC  3 | VC  3 | VC  3 | VC  3 | VC  3 | VC  3 | 2.82 | A |
| T5S3 | Learning from other specialties gives a greater insight into the working of other disciplines | VC  3 | VC  3 | VC  3 | VC  3 | VC  3 | VC  3 | VC  3 | VC  3 | VC  3 | VC  3 | VC  3 | VC  3 | VC  3 | VC  3 | VC  3 | VC  3 | VC  3 | 3 | A |
| T5S4 | I share mutual knowledge with other specialties to handle clinical cases effectively | VC  3 | VC  3 | VC  3 | R  2 | VC  3 | VC  3 | VC  3 | VC  3 | VC  3 | VC  3 | VC  3 | VC  3 | VC  3 | VC  3 | VC  3 | VC  3 | VC  3 | 2.94 | A |
|  | **Theme 6: Intraprofessional collaborative leadership** | | | | | | | | | | | | | | | | | | | |
| T6S1 | I help my colleagues to address conflict with other disciplines effectively | VC  3 | VC  3 | VC  3 | V  C  3 | VC  3 | VC  3 | VC  3 | VC  3 | R  2 | VC  3 | VC  3 | VC  3 | VC  3 | VC  3 | VC  3 | VC  3 | VC  3 | 2.94 | A |
| T6S2 | I can motivate and influence colleagues and build teams | VC  3 | VC  3 | VC  3 | VC  3 | VC  3 | VC  3 | VC  3 | VC  3 | VC  3 | VC  3 | VC  3 | VC  3 | VC  3 | VC  3 | VC  3 | VC  3 | VC  3 | 3 | A |
| T6S3 | I can coordinate and plan collaborative meetings and processes | VC  3 | VC  3 | VC  3 | VC  3 | VC  3 | VC  3 | VC  3 | VC  3 | VC  3 | VC  3 | VC  3 | VC  3 | VC  3 | VC  3 | VC  3 | VC  3 | VC  3 | 3 | A |
| T6S4 | I take responsibility of developing patient care plans when working in multidisciplinary teams | R  2 | VC  3 | VC  3 | VC  3 | VC  3 | VC  3 | VC  3 | VC  3 | VC  3 | VC  3 | VC  3 | VC  3 | VC  3 | VC  3 | VC  3 | VC  3 | VC  3 | 2.94 | A |
| T6S5 | I try to create a conducive environment where inputs from various specialties are encouraged and utilized towards patient centered care | VC  3 | VC  3 | VC  3 | VC  3 | VC  3 | R  2 | VC  3 | VC  3 | VC  3 | VC  3 | VC  3 | VC  3 | VC  3 | VC  3 | VC  3 | VC  3 | VC  3 | 2.94 | A |

VC= Very clear

R= Item needs revision

NC= Not clear

CCA= Content clarity average,

A= Accepted

AM= Accepted after Modification

D= Deleted

## Annexure IV: Cognitive Pretesting for Response Process Validity

| **Sr No** | **Main construct** | **Item** | **Qualitative Analysis** | **Code** |
| --- | --- | --- | --- | --- |
| 1 | **Roles/responsibilities for Intraprofessional collaborative practice** | I can share my learning with residents of other disciplines with ease when required | “Yes, I understand this item and I feel I can easily share my learning experiences with other residents without any difficulty when required” | No change |
| 2 |  | I welcome the opportunity to work with other health professionals in small group learning activities for patient care | “Yes, the statement is clear. I feel happy to be part of activities related to patient care involving other specialty residents. It broadens my horizon” | No change |
| 3 |  | I am aware of my role as well as my limitations in patient care | “Yes, I understand this. I am aware of how I have to go about in patient care and what are my limitations” | No change |
| 4 |  | I can approach other professionals in different disciplines for their particular expertise for multidisciplinary patient care | “I think it means that I don’t feel any hinderances in approaching residents of other disciplines. Or are you asking about all professionals?” | Minor problem-rephrase  I can conveniently approach residents in other specialties for their particular expertise for multidisciplinary patient care |
| 5 |  | I consistently give feedback to other residents in my setting about a relevant case when required | “Feedback is very important, and one should give it in an appropriate manner. This leads to better patient care. I understand this statement emphasizes the importance of feedback.” | No change |

| 6 |  | I take feedback from my colleagues in other specialties positively | “Yes, I understand this stem. One should be happy to receive feedback as well.” | No change |
| --- | --- | --- | --- | --- |
| 7 | **Intraprofessional collaborative communication** | I frequently communicate with my colleagues from other disciplines | “I think this statement refers to the frequency of our communication and that it’s a routine that we communicate.” | No change |
| 8 |  | I understand when and what has to be communicated | “Does this mean at what time I communicate? And what exactly should I ask? Yes, I think I am aware of what to do. This needs to be clearer” | Minor problem-rephrase  I am fully aware when and what has to be communicated to seek help from fellow residents in other disciplines. |
| 9 |  | I can assess the urgency of the situation and communicate timely with colleagues according to the medical condition of patient. | “Yes, I am being asked about if I know when to initiate a red call and when a yellow call. This is about timing according to severity of patient condition.” | No change |
| 10 |  | I am well versed with principles of written communication (should be timely, precise and in appropriate language) | “Yes, we are taught multiple times by our supervisor about how to frame a referral. It is must to know how to refer timely and be precise in a written referral.” | No change |
| 11 |  | I am fully aware of the extent to which I have to seek help and involvement of fellow residents from other disciplines in a particular case. | “This is asking about my knowledge about how much I should involve another colleague in dealing with a specific patient” | No change |
| 12 | **Intraprofessional collaborative team-based care and networking** | I know many of the other residents personally | “Yes, I understand. We do know other residents and have friendships and good rapport with some.” | No change |
| 13 |  | I know the workplace resources and limitations of other specialties | “Yes, the statement is clear. We all have limitations in resources and workplace problems and problems faced by others should be understood.” | No change |
| 14 |  | I understand the perspective (viewpoint) of other disciplines’ residents in patient care | “I understand the statement. Different specialties have different viewpoints about a particular case, and I should be able to understand the view.” | No change |
| 15 |  | I understand team dynamics and power relations | “I don’t understand it clearly. Power dynamics means somebody is more authoritative in a team? Yes, we face that problem. I think more appropriate to ask power dynamics in a team.” | Minor problem-rephrase  I understand power dynamics in a team |
| 16 | **Values/ethics for intraprofessional collaborative practice** | I respect the roles, expertise and task distribution of other team members | It is a very clear statement. It is asking about my respect for the role of other members in the team. | No change |
| 17 |  | I respect the values of fellow residents related to patients’ outcome | What exactly is meant by values of other residents? If this means opinions then I agree that it is essential to respect the opinions of others and consider them for better patient care. | Minor problem-rephrase  I respect the opinions of fellow residents related to patients’ care. |
| 18 |  | I am willing to cooperate with other residents without any preconceived notions | “Yes, I understand. One can only cooperate if one goes into a discussion with an open mind and has no preconceived ideas.” | No change |
| 19 |  | I am not prejudiced against other specialties | “I think it more or less means the same. Collaboration cannot occur if we are already prejudiced about something.” | Delete? |
| 20 |  | I have the ability to look beyond my own position and task to get a wider picture | “Yes, I think it means that I understand their viewpoint, and this will help me understand things better regarding a patient.” | No change |
| 21 |  | I work from a patient centered perspective in my practice | “This is pretty simple. The basic purpose of all this is that the benefit of the patient be central to what we do in a team.” | No change |
| 22 | **Sharing of mutual knowledge for IPCP** | I can willingly sacrifice a degree of autonomy (accept somebody else’s decision) to support cooperative problem solving. | “Yes, I understand. It is about the ability to let go my own decision and accept other persons decision like it involves being flexible.” | No change |
| 23 |  | I utilize formal and informal channels for problem-solving with my colleagues from other disciplines | “I think it means that I use the official referral system as well as my personal connections to involve other residents” | No change |
| 24 |  | Learning from other specialties gives a greater insight into the working of other disciplines | “Yes, no doubt. Learning from other adds a greater understanding to the case we are dealing.” | No change |
| 25 |  | I share mutual knowledge with other specialties to handle clinical cases effectively | “What is meant by mutual knowledge? I think it is about sharing my knowledge with others.” | Minor-change-rephrase  I share my knowledge... |

| 26 | **Intraprofessional collaborative leadership** | I help my colleagues to address conflict with other disciplines effectively. | “This means that whenever there is a team, there are bound to be conflicts, so one must have ability to address such conflicts.” | No change |
| --- | --- | --- | --- | --- |
| 27 |  | I can motivate and influence colleagues and build teams | “This is about my ability to bring together other people together for a task related to patient care. It’s a clear statement.” | No change |
| 28 |  | I can coordinate and plan collaborative meetings and processes | “Yes, this is pretty clear. It is about coordinating a multi-disciplinary meeting.” | No change |
| 29 |  | I take responsibility of developing patient care plans when working in multidisciplinary teams | “Yes, I understand. Collaboration is good but somebody has to take responsibility of the task and coordinate with other fellows. I think I can do it.” | No change |
| 30 |  | I try to create a conducive environment where inputs from various specialties are encouraged and utilized towards patient centered care | “This is a question about if I make efforts to keep a friendly environment where everybody’s input is valued and encouraged.” | No change |

Annexure V: Reliability statistics of Individual Items

| **Item-Total Statistics** | | | | |
| --- | --- | --- | --- | --- |
|  | Scale Mean if Item Deleted | Scale Variance if Item Deleted | Corrected Item-Total Correlation | Cronbach's Alpha if Item Deleted |
| I can share my learning with residents of other disciplines with ease when required | 117.5111 | 143.127 | .528 | .935 |
| I welcome the opportunity to work with other health professionals in small group learning activities for patient care | 117.3587 | 145.438 | .513 | .935 |
| I am aware of my role as well as my limitations in patient care | 117.2776 | 144.457 | .572 | .935 |
| I can conveniently approach residents in other specialties for their particular expertise for multidisciplinary patient care | 117.6634 | 142.948 | .504 | .935 |
| I consistently give feedback to other residents in my setting about a relevant case when required | 117.8305 | 143.185 | .473 | .936 |
| I take feedback from my colleagues in other specialties positively | 117.6806 | 143.351 | .486 | .936 |
| My colleagues from other disciplines and I frequently communicate regarding patient care | 117.4251 | 143.659 | .621 | .934 |
| I am fully aware when and what has to be communicated to seek help from fellow residents in other disciplines. | 117.3661 | 144.686 | .580 | .934 |
| I can communicate timely according to the urgency of medical condition of patient. | 117.3808 | 143.389 | .572 | .934 |
| I am well versed with principles of written communication (should be timely, precise and in appropriate language) | 117.4300 | 142.940 | .631 | .934 |
| I discuss with other disciplines the degree to which each of us should be involved in a particular case | 117.4324 | 142.857 | .673 | .933 |
| I know many of the other residents personally | 117.9189 | 142.380 | .467 | .936 |
| I know the workplace, resources and limitations of other specialties | 117.8108 | 142.435 | .551 | .935 |
| I understand the perspective (viewpoint) of other disciplines’ residents in patient care | 117.7641 | 143.186 | .576 | .934 |
| I understand power dynamics in a team | 117.5135 | 143.265 | .629 | .934 |
| I respect the roles, expertise and task distribution of other team members | 117.3170 | 144.552 | .650 | .934 |
| I respect the opinions of fellow residents related to patients’ outcome | 117.3145 | 145.024 | .619 | .934 |
| I am willing to cooperate with other residents without any preconceived notions | 117.3415 | 144.728 | .602 | .934 |
| I have the ability to look beyond my own position and task to get a wider picture | 117.5283 | 143.358 | .604 | .934 |
| I work from a patient centered perspective in my practice | 117.3538 | 144.983 | .582 | .934 |
| I can willingly sacrifice a degree of autonomy (accept somebody else’s decision) to support cooperative problem solving. | 117.6953 | 144.365 | .466 | .936 |
| I utilize formal and informal channels for problem-solving with my colleagues from other disciplines | 117.6634 | 144.569 | .506 | .935 |
| Learning from other specialties gives a greater insight into the working of other disciplines | 117.3735 | 145.619 | .510 | .935 |
| I share my knowledge with other specialties to handle clinical cases effectively | 117.4791 | 142.871 | .668 | .933 |
| I help my colleagues to address conflict with other disciplines effectively. | 117.6216 | 143.763 | .591 | .934 |
| I can motivate and influence colleagues and build teams | 117.6192 | 143.379 | .571 | .934 |
| I can coordinate and plan collaborative meetings and processes | 117.7101 | 142.443 | .577 | .934 |
| I take responsibility of developing patient care plans when working in multidisciplinary teams | 117.6634 | 141.569 | .598 | .934 |
| I try to create a conducive environment where inputs from various specialties are encouraged and utilized towards patient centered care | 117.5946 | 141.848 | .665 | .933 |

**Annexure VI: Final Instrument**

This self-assessment instrument contains 29 items relating to a range of statements depicting Intraprofessional Collaborative Practices in postgraduate residents. This is an online instrument and can be used by postgraduate residents of multiple specialties like Medicine & Allied, Surgery & Allied, Gynecology and Pediatrics to self-assess their collaboratives practices while working in tertiary care hospitals. You can score the items on a 5-point Likert scale ranging from strongly agree to strongly disagree according to your current practices.

**Scoring the IPCP-R**

Items should be scored as follows:

5 Strongly Agree

4 Agree

3 Neither agree nor disagree

2 Disagree

1 Strongly disagree

The 29-item IPCP-R has a maximum score of 145 indicating excellent intraprofessional collaborative practices. A score of 29 is the minimum.

The instrument has six subscales:

- Roles/responsibilities for IPCP
- Intraprofessional collaborative communication
- Intraprofessional collaborative team-based care
- Values/ethics for IPCP
- Sharing of mutual knowledge for IPCP
- Intraprofessional collaborative leadership

| **Sr No** | **Items** | **Strongly Agree** | **Agree** | **Neither Agree nor Disagree** | **Disagree** | **Strongly disagree** |
| --- | --- | --- | --- | --- | --- | --- |
| **Theme 1: Roles/responsibilities for Intraprofessional collaborative practice** | | | | | | |
| T1S1 | I can share my learning with residents of other disciplines with ease when required |  |  |  |  |  |
| T1S2 | I welcome the opportunity to work with other health professionals in small group learning activities for patient care |  |  |  |  |  |
| T1S3 | I am aware of my role as well as my limitations in patient care |  |  |  |  |  |
| T1S4 | I can conveniently approach residents in other specialties for their particular expertise for multidisciplinary patient care |  |  |  |  |  |
| T1S5 | I consistently give feedback to other residents in my setting about a relevant case when required |  |  |  |  |  |
| T1S6 | I take feedback from my colleagues in other specialties positively |  |  |  |  |  |
|  | ***6 items/maximum score of 30 for this sub-scale*** | | | | | |
| **Theme 2: Intraprofessional collaborative communication** | | | | | | |
| T2S1 | My colleagues from other disciplines and I frequently communicate regarding patient care |  |  |  |  |  |
| T2S2 | I am fully aware when and what has to be communicated to seek help from fellow residents in other disciplines. |  |  |  |  |  |
| T2S3 | I can communicate timely according to the urgency of medical condition of patient. |  |  |  |  |  |
| T2S4 | I am well versed with principles of written communication (should be timely, precise and in appropriate language) |  |  |  |  |  |
| T2S5 | I discuss with other disciplines the degree to which each of us should be involved in a particular case |  |  |  |  |  |
|  | ***5 items/maximum score of 25 for this sub-scale*** | | | | | |
| **Theme 3: Intraprofessional collaborative team-based care and networking** | | | | | | |
| T3S1 | I know many of the other residents personally |  |  |  |  |  |
| T3S2 | I know the workplace, resources and limitations of other specialties |  |  |  |  |  |
| T3S3 | I understand the perspective (viewpoint) of other disciplines’ residents in patient care |  |  |  |  |  |
| T3S4 | I understand power dynamics in a team |  |  |  |  |  |
|  | ***4 items/maximum score of 20 for this sub-scale*** | | | | | |
| **Theme 4: Values/ethics for intraprofessional collaborative practice** | | | | | | |
| T4S1 | I respect the roles, expertise and task distribution of other team members |  |  |  |  |  |
| T4S2 | I respect the opinions of fellow residents related to patients’ outcome |  |  |  |  |  |
| T4S3 | I am willing to cooperate with other residents without any preconceived notions |  |  |  |  |  |
| T4S4 | I have the ability to look beyond my own position and task to get a wider picture |  |  |  |  |  |
| T4S5 | I work from a patient centered perspective in my practice |  |  |  |  |  |
|  | ***5 items/maximum score of 30 for this sub-scale*** | | | | | |
| **Theme 5: Sharing of mutual knowledge for IPCP** | | | | | | |
| T5S1 | I can willingly sacrifice a degree of autonomy (accept somebody else’s decision) to support cooperative problem solving. |  |  |  |  |  |
| T5S2 | I utilize formal and informal channels for problem-solving with my colleagues from other disciplines |  |  |  |  |  |
| T5S3 | Learning from other specialties gives a greater insight into the working of other disciplines |  |  |  |  |  |
| T5S4 | I share my knowledge with other specialties to handle clinical cases effectively |  |  |  |  |  |
|  | ***4 items/maximum score of 20 for this sub-scale*** | | | | | |
| **Theme 6: Intraprofessional collaborative leadership** | | | | | | |
| T6S1 | I help my colleagues to address conflict with other disciplines effectively. |  |  |  |  |  |
| T6S2 | I can motivate and influence colleagues and build teams |  |  |  |  |  |
| T6S3 | I can coordinate and plan collaborative meetings and processes |  |  |  |  |  |
| T6S4 | I take responsibility of developing patient care plans when working in multidisciplinary teams |  |  |  |  |  |
| T6S5 | I try to create a conducive environment where inputs from various specialties are encouraged and utilized towards patient centered care |  |  |  |  |  |
|  | ***5 items/maximum score of 25 for this sub-scale*** | | | | | |
